# Synthesis and Cytotoxicity against K562 Cells of 3-*O*-Angeloyl-20-*O*-acetyl Ingenol, a Derivative of Ingenol Mebutate

**DOI:** 10.3390/ijms17081348

**Published:** 2016-08-19

**Authors:** Ming Liu, Fangling Chen, Rilei Yu, Weiyi Zhang, Mei Han, Fei Liu, Jing Wu, Xingzeng Zhao, Jinlai Miao

**Affiliations:** 1 Key Laboratory of Marine Drugs, Ministry of Education, School of Medicine and Pharmacy, Ocean University of China, Qingdao 266003, China; Chenfangling0410@hotmail.com (F.C.); rileiyu2010@hotmail.com (R.Y.); 13070868219@163.com (W.Z.); 6151504014@vip.jiangnan.edu.cn (J.W.); 2 Department of Pharmacology, Medical College Qingdao University, Qingdao 266071, China; maviswga@163.com; 3 Institute of Botany, Jiangsu Province and Chinese Academy of Sciences (Nanjing Botanical Garden, Mem Sun Yat-sen), Nanjing 210014, China; liufeiseu@163.com (F.L.); zhaoxingzeng@hotmail.com (X.Z.); 4 Key Laboratory of Marine Bioactive Substance, The First Institute of Oceanography, State Oceanic Administration, Qingdao 266061, China

**Keywords:** 3-*O*-angeloyl-20-*O*-acetyl ingenol, PEP008, 20-*O*-acetyl-ingenol-3-angelate, ingenol mebutat, apoptosis, chronic myeloid leukemia

## Abstract

Ingenol mebutate possesses significant cytotoxicity and is clinically used to treat actinic keratosis. However, ingenol mebutate undergoes acyl migration which affects its bioactivity. Compound 3-*O*-angeloyl-20-*O*-acetyl ingenol (AAI, also known as 20-*O*-acetyl-ingenol-3-angelate or PEP008) is a synthetic derivative of ingenol mebutate. In this work, we report the AAI synthesis details and demonstrate AAI has higher cytotoxicity than ingenol mebutate in a chronic myeloid leukemia K562 cell line. Our data indicate that the increased activity of AAI originates from the improved intracellular stability of AAI rather than the increased binding affinity between AAI and the target protein protein kinase Cδ (PKCδ). AAI inhibits cell proliferation, induces G2/M phase arrest, disrupts the mitochondrial membrane potential, and stimulates apoptosis, as well as necrosis in K562 cells. Similar to ingenol mebutate, AAI activates PKCδ and extracellular signal regulated kinase (ERK), and inactivates protein kinase B (AKT). Furthermore, AAI also inhibits JAK/STAT3 pathway. Altogether, our studies show that ingenol derivative AAI is cytotoxic to K562 cells and modulates PKCδ/ERK, JAK/STAT3, and AKT signaling pathways. Our work suggests that AAI may be a new candidate of chemotherapeutic agent.

## 1. Introduction

**Figure 1:**
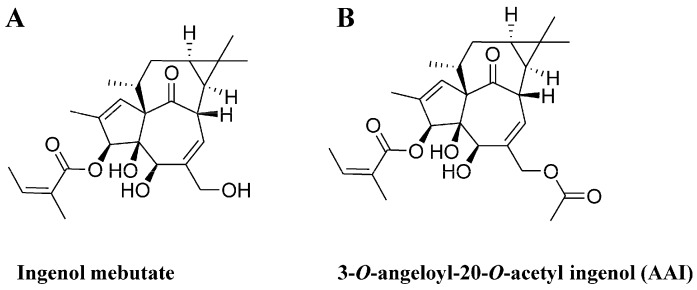
Chemical structures of ingenol mebutate (**A**) and 3-*O*-angeloyl-20-*O*-acetyl ingenol (**B**).

Ingenol mebutate (also called PEP005 or ingenol 3-anglate, Figure 1A) is a hydrophobic diterpene ester which is isolated from the sap of the plant *Euphorbia peplus*. Ingenol mebutate has potent anti-cancer effects both in vitro and in vivo, and is used as the active ingredient in Picato^®^, a new drug for the treatment of actinic keratosis [1,2]. Preclinical studies demonstrate that ingenol mebutate has potent activities against a number of leukemic cell lines, stimulating both apoptosis and necrosis. It is believed that ingenol mebutate is a potent activator of PKCδ [3]. PKCδ belongs to the protein kinase C (PKC) family of signaling isoenzymes which regulates cell proliferation, differentiation, and apoptosis. Depending on the cell type, PKCδ can function as a tumor suppressor or a pro-apoptotic factor and regulate cell proliferation and survival functions. Induction of PKCδ has been previously shown in promyelocytic leukemia cells, and PKCδ regulates eIF2α through induction of PKR in acute myeloid leukemia cells [4]; other studies have shown that activation of PKCδ can lead to ERK1/2 phosphorylation [3]. In recent years, PKCδ-involved signaling pathways have been considered as targets of several novel anti-cancer agents and the ability of PKC activators to induce apoptosis in a wide range of leukemia-derived cell lines has made them attractive targets for the development of anti-leukemic drugs [5,6].

Previous investigations have revealed that the 5-hydroxyl group of ingenol mebutate is necessary for its bioactivity. However, the 3-angelate undergoes acyl migration in aqueous solution to form 5- and 20-mono-angelates (1:18), and this migration thus affects its bioactivity [7]. In the search of ingenol mebutate analogs with improved chemical stability and efficiency, we synthesized 3-*O*-angeloyl-20-*O*-acetyl ingenol (AAI, Figure 1B) by acetylation at the 20 position of ingenol mebutate, which may improve compound stability and enhance its hydrophobicity. AAI has been previously named as 20-*O*-acetyl-ingenol-3-angelate or PEP008 (PubChem CID: 14136933) and is sensitive against cancer cells derived from melanoma, breast cancer, and colon cancer [8]. Considering AAI is a structural derivative of ingenol mebutate, which shows a wide range of anti-cancer spectra, we hypothesized that AAI could be cytotoxic to other cell lines, such as the leukemia, and the increased stability and hydrophobicity could enhance its bioactivity.

Here, we reported the synthesis details of AAI, and the underlying molecular mechanisms of its cytotoxicity. We demonstrated that AAI inhibited cell proliferation, disturbed the cell cycle distribution, and stimulated apoptosis in K562 cells. We also illustrated that AAI modulated multiple signaling pathways, including activating PKCδ/ERK pathway, inhibiting protein kinase B AKT, and on JAK/STAT3 pathway, indicating the potential applications of AAI in anti-cancer research and development.

## 2. Results

### 2.1. The Synthesis of 3-O-Angeloyl-20-O-acetyl ingenol (AAI)

**Scheme 1:**
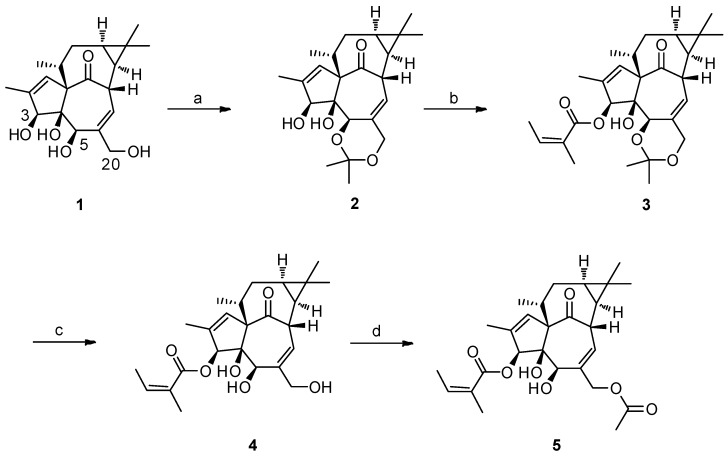
Synthesis of 3-*O*-angeloyl-20-*O*-acetyl ingenol. (**a**) PTSA·H_2_O, acetone; (**b**) angelic anhydride, lithium hexamethyldisilazide (LHMDS), tetrahydrofuran (THF); (**c**) 1% HCl, MeOH; and (**d**) Ac_2_O, pyridine.

Considering that the 3-angelate group in ingenol mebutate undergoes acyl migration to form inactive 5- and 20-mono-angelates, we made structural changes of ingenol mebutate by 20-acetylation to obtained AAI (Scheme 1). The established synthesis route was started from ingenol, followed by four steps, as shown in Scheme 1, each step with high yield. The structure of the target compound **5** was confirmed by the nuclear magnetic resonance (NMR, Supplementary Materials Figure S1) data: ^1^H-NMR (CDCl_3_, 400 MHz): δ 0.69–0.70 (m, 1H); 0.93–0.98 (m, 4H); 1.05–1.08 (d, 3H); 1.25–1.40 (m, 4H); 1.74–1.78 (m, 3H); 1.80 (s, 3H); 1.92 (s, 3H); 2.00–2.02 (t, 3H); 2.06 (s, 3H); 2.21–2.24 (t, 1H); 2.49 (s, 1H); 3.89 (s, 1H); 4.07–4.11 (q, 1H); 4.46–4.78 (dd, 1H); 5.55 (s, 1H); 6.04 (s, 1H); 6.12–6.14 (m, 1H); 6.16–6.19 (m, 1H).

### 2.2. AAI Inhibits Cell Proliferation

**Figure 2:**
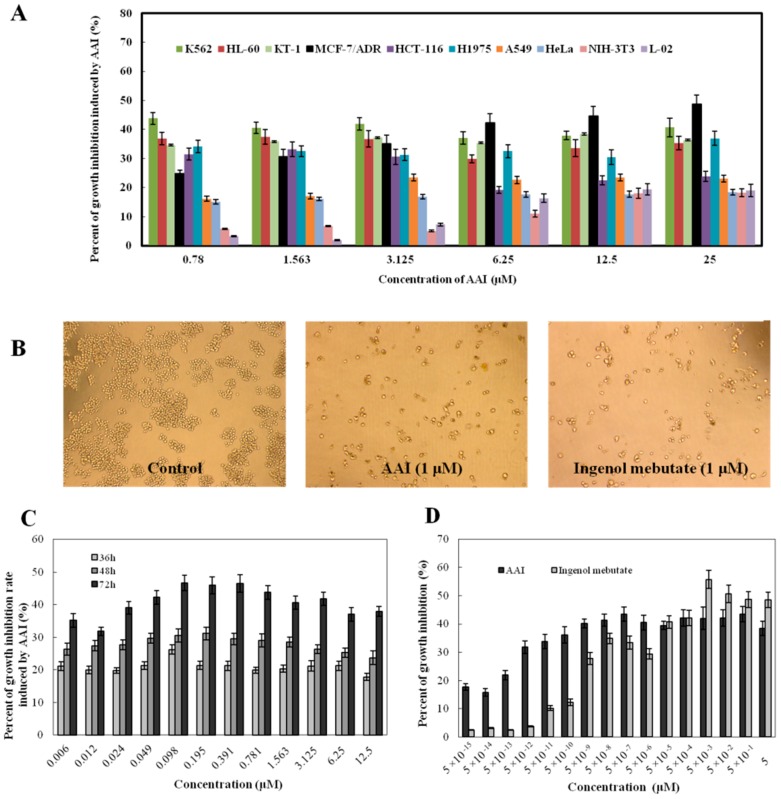
(**A**) Cytotoxicity of AAI assayed by 3-(4,5-dimethyl-2-thiazolyl)-2,5-diphenyl-2-*H*-tetrazolium bromide (MTT) methods. Tested cells in 96-well plates were treated with (0–25 μM) AAI for 72 h, and then the viability was evaluated by MTT assay, as described in the Materials and Methods section; (**B**) AAI decreases the population of K562 cells in a comparable potency of ingenol mebutate. Cells in 96-well plates were treated with 1 μM AAI or ingenol mebutate for 72 h, and then the cell density was photographed (×200); (**C**) growth inhibition of AAI against K562 cells. Cells were treated with indicated concentrations of AAI for 36, 48, and 72 h, and then the viability was evaluated by MTT assay; and (**D**) AAI shows stronger growth inhibition than ingenol mebutate at low concentrations. Cells were treated with indicated concentrations of AAI or ingenol mebutate for 72 h, and then cell viability was evaluated by MTT assay.

To evaluate the cytotoxicity of AAI in vitro, we tested its effects on cell proliferation using several human tumor cell lines (human myeloid leukemia K562, HL-60, and KT-1; adriamycin-resistant human breast carcinoma MCF-7/ADR, a human breast cancer cell line resistant to adriamycin; human colorectal carcinoma HCT-116; human lung adenocarcinoma H1975, A549; and human cervical carcinoma HeLa), as well as two normal cell lines (human normal liver cells L-02 and fibroblast cells NIH-3T3), and the growth inhibition rates are shown in Figure 2A. AAI (0.78–25 μM) showed different cytotoxicity on the selected cell lines. K562, MCF-7/ADR, KT-1, and HL-60 cells were relatively more sensitive to AAI. At 1 μM concentration, AAI inhibited K562 cells growth at a similar level as ingenol mebutate (Figure 2B). However, the observed cytotoxicity of AAI was not concentration-dependent (Figure 2A), but obviously time-dependent (Figure 2C). It is interesting that AAI showed growth inhibition against K562 cells even at very low concentration (5 × 10^−15^ to 5 × 10^−10^ μM), and the inhibition effects were much more potent than ingenol mebutate (Figure 2D). Under such low concentrations (5 × 10^−15^ to 5 × 10^−10^ μM), AAI did not show any cytotoxicity to other tested cell lines (data not shown), thus K562 cells were selected for the following studies.

### 2.3. AAI Induces G2/M Phase Arrest, Apoptosis, and Necrosis in K562 Cells

**Figure 3:**
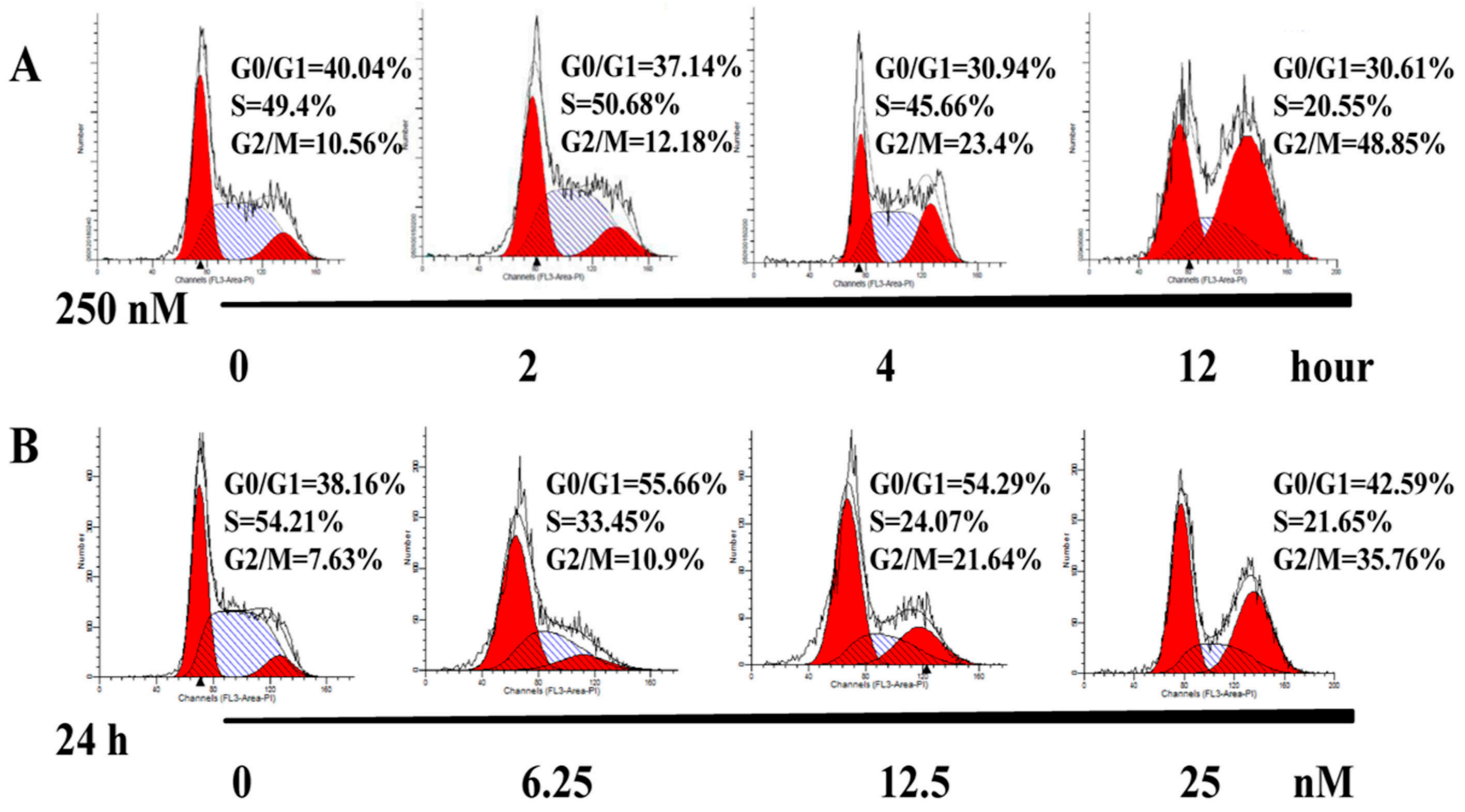

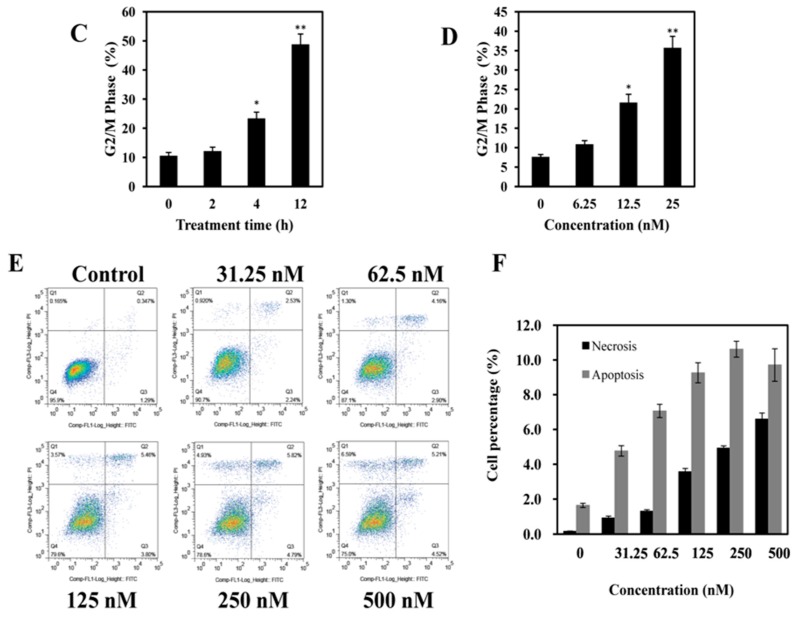
AAI induces G2/M phase arrest, apoptosis, and necrosis in K562 cells. (**A**) AAI time-dependently arrests K562 cells at G2/M phase. K562 cells were treated with 250 nM AAI for 0, 2, 4 and 12 h. Then, cells were collected, fixed, digestion with RNase A, and stained by PI. The DNA contents of the cells were determined with the Aria FACS flow cytometry system; red: G0/G1 or G2/M phase, blue: S phase; (**B**) K562 cells were treated with AAI (0–25 nM) for 24 h, and then the cell cycle distribution was analyzed; red: G0/G1 or G2/M phase, blue: S phase; (**C**) histograms show the percentage of cells distributed in G2/M phase after treated with 250 nM AAI for 0, 2, 4 and 12 h; * *p* < 0.05, ** *p* < 0.01 versus control; (**D**) histograms show the percentage of cells distributed in G2/M phase after treated with AAI (0–25 nM) for 24 h; * *p* < 0.05, ** *p* < 0.01 versus control; (**E**) AAI induces both apoptosis and necrosis in K562 cells. K562 cells were untreated or treated with 31.25, 62.5, 125, 250 and 500 nM AAI for 18 h, and then the cells were double-stained with Annexin V-FITC/PI and analyzed by flow cytometry. The percentage of Annexin V-FITC positive cells and/or PI positive cells is indicated; and (**F**) histogram shows the percentage of necrosis and apoptosis in K562 cells induced by AAI.

Cell cycle arrest usually contributes to the growth inhibition; we, thus, analyzed the cell cycle distribution in K562 cells. As shown in Figure 3A, the percentage of K562 cells in G2/M phase of the untreated group was 10.56%; after treatment with AAI (250 nm) for 2, 4, and 12 h, it increased to 12.18%, 23.4%, and 48.85%, respectively, in a time-dependent manner (Figure 3C). Similarly, when treated with different concentration (0–25 nM) of AAI for 24 h, K562 cells also showed a marked G2/M phase arrest (Figure 3B,D). These results indicated that AAI could arrest K562 cells at G2/M phase, which may contribute to AAI-induced growth inhibition.

To evaluate whether apoptosis and necrosis were account for AAI-induced growth inhibition in K562 cells, we performed Annexin V-FITC/PI double-staining assay. After incubated with AAI (0–500 nM) for 18 h, both the percentage of Annexin V-FITC positive and PI positive cells increased (Figure 3E), indicating AAI induced both apoptosis and necrosis in K562 cells. The percentage of apoptotic cells increased from 1.64% in the control group to 10.6% and 9.7% when treatment with 250 and 500 nM AAI, respectively. In addition, the percentage of the necrosis cells in the control group was 0.16%; after AAI (500 nM) treatment, the percentage increased to 6.59% (Figure 3F). Although the percentage of both apoptosis and necrosis is not very high under the present experimental conditions, considering its high chemical structural similarity with ingenol mebutate and the observed similar apoptosis/necrosis-inducing activity, our present results indicated that AAI could also stimulate both apoptosis and necrosis in K562 cells.

### 2.4. AAI Disturbs Mitochondrial Membrane Potential (MMP) in K562 Cells

**Figure 4:**
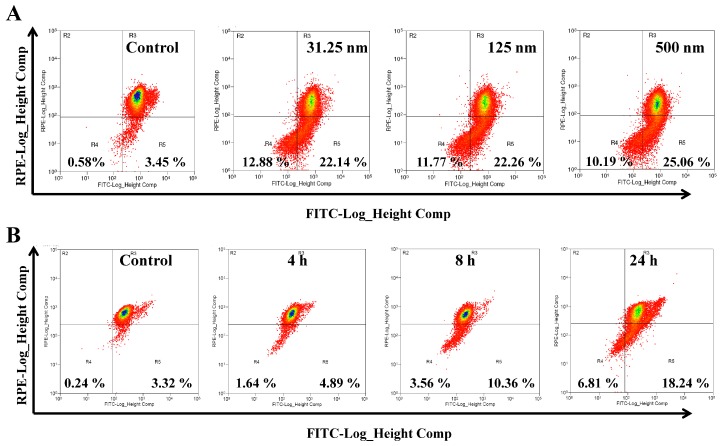
AAI induces the loss of MMP in K562 cells. (**A**) Treatment with AAI (0–500 nM) for 18 h disrupts the MMP in K562 cells. The scatter plot of the flow cytometry analysis shows the distribution of JC-1 aggregates and JC-1 monomers; and (**B**) AAI at 250 nM time-dependently induces the loss of MMP in K562 cells.

The disruption of MMP is an important event in chemical agents-induced apoptosis. To further confirm the AAI-induced apoptosis, we tested the effect of AAI on MMP in K562 cells using a JC-1 probe. The loss of MMP was generally reflected by the increased green fluorescence from JC-1 monomers, as well as the decreased red fluorescence from JC-1 aggregates. As shown in Figure 4A, when treated with different concentrations (0–500 nM) of AAI, there were significant increases in green JC-1 monomers, and decreases in red JC-1 aggregates, indicating the loss of cell MMP. Similarly, AAI (250 nm) time-dependently increased the green fluorescence and decreased the red fluorescence (Figure 4B), further confirming the disruption of MMP in K562 cells. These results demonstrated that AAI disrupted the mitochondrial function which confirmed the AAI-induced apoptosis in K562 cells.

### 2.5. AAI Modulates Multiple Key Signaling Pathways in K562 Cells

**Figure 5:**
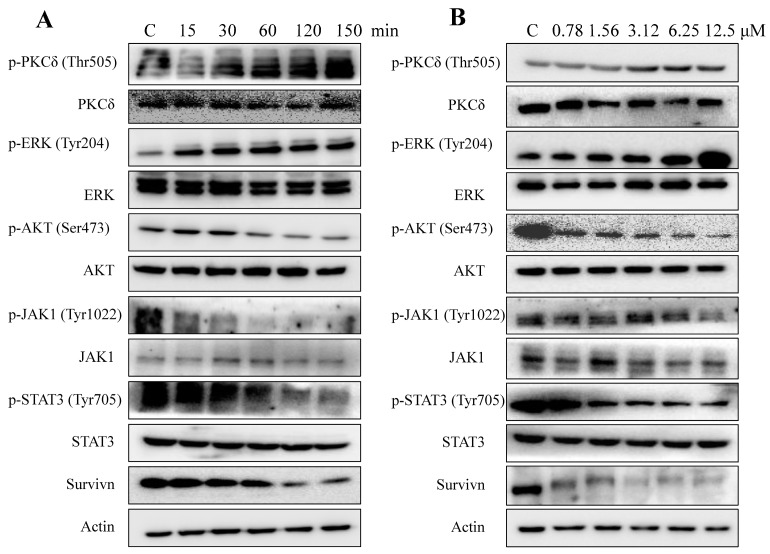
AAI modulates multiple signaling pathways in K562 cells. (**A**) AAI induces time-dependent activation of PKC and ERK, inactivation of AKT, and inhibition on phosphorylation of JAK and STAT3, and decreases the expression level of surviving; and (**B**) different concentrations (0–12.5 μM) of AAI treated for 24 h activate PKCδ and ERK, inactivate AKT, inhibit JAK/STAT3 pathway, and decrease the expression level of survivin.

To illustrate the intracellular apoptosis- and survival-related signaling events triggered by AAI, we tested its effects on several signaling molecules. We found that AAI-treated K562 cells had much higher expression levels of p-PKCδ and p-ERK compared with the control cells, without any obvious changes in the total levels of PKCδ and ERK. This result indicated the activating of PKCδ/ERK pathway by AAI (Figure 5A). Activation of AKT has been considered as one of the main signaling events in the survival of K562 cells. In the present studies, we found that activation of AKT was inhibited by AAI (250 nM) in a time-dependent manner, with no effect on the total AKT level. Moreover, our results also showed that AAI could time-dependently inhibit the activation of JAK/STAT3 pathway; AAI could inactivate both p-JAK and p-STAT3, which play vital roles in leukemogenesis and are attractive targets for therapeutic agents. As the downstream product of JAK/STAT3, survivin protein expression level decreased, correspondingly. However, p-STAT2/5 remained unchanged (Supplementary Materials, Figure S2). Similarly, with longer treatment period of 24 h, AAI also activated the PKCδ/ERK pathway, inactivated AKT, inhibited the activation of JAK/STAT3, and downregulated the expression level of survivin (Figure 5B). These results showed that AAI could modulate multiple signaling pathways related to leukemogenesis.

### 2.6. AAI and Ingenol Mebutate Bind PKCδ in a Similar Manner

**Figure 6:**
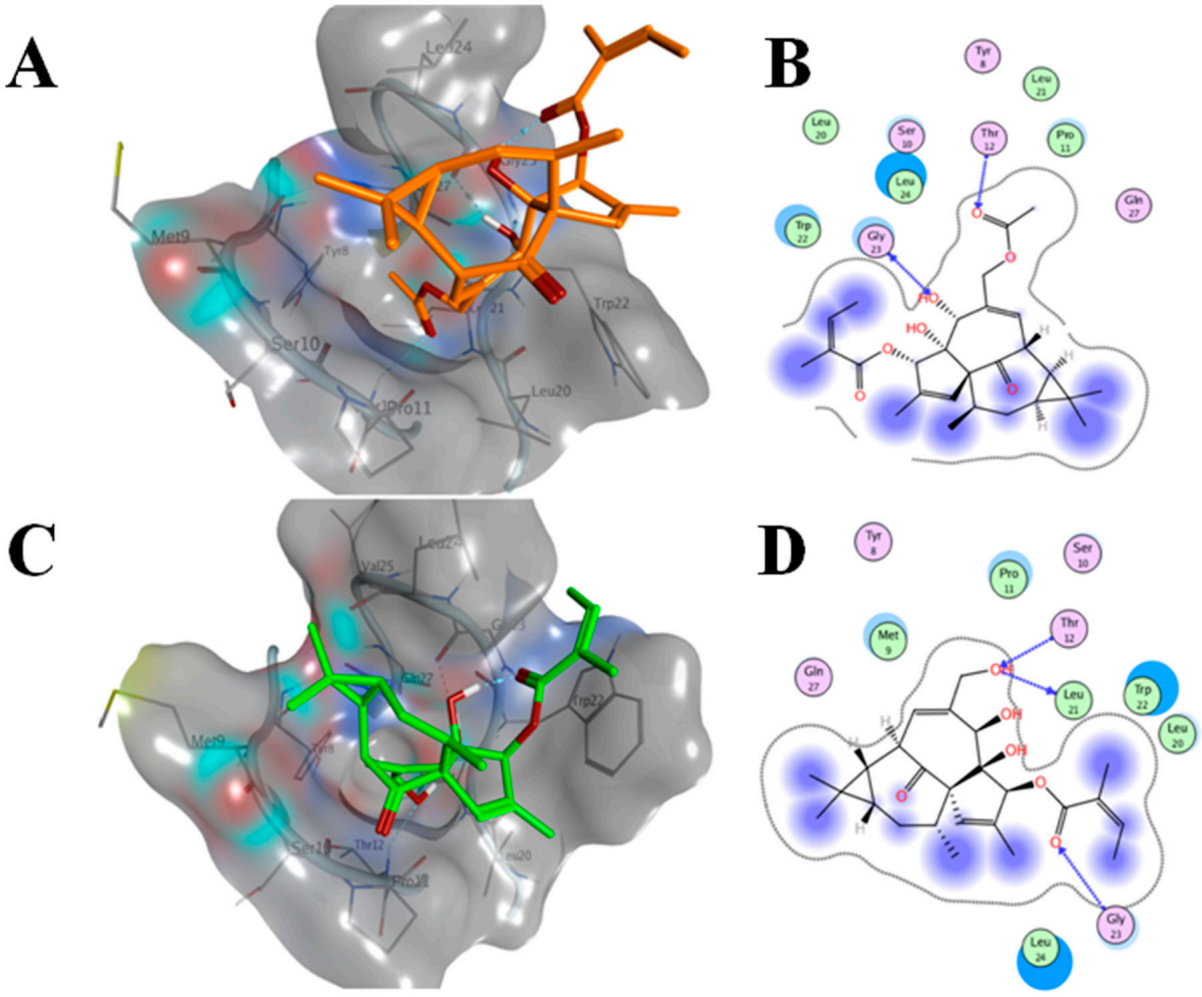

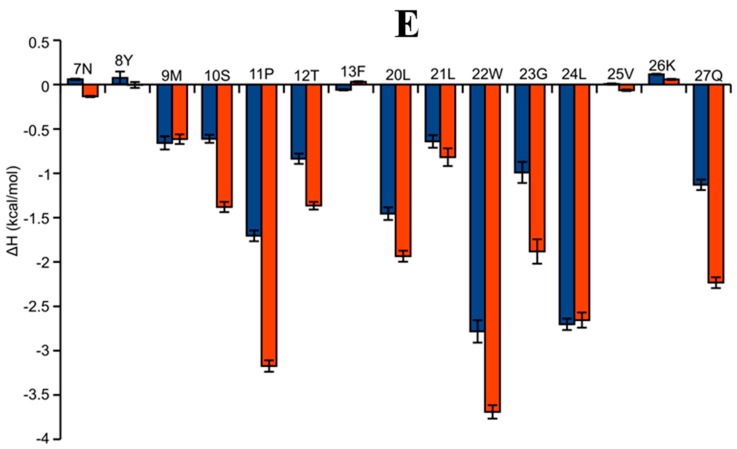
Binding modes of AAI (orange, **A**) and ingenol mebutate (green, **C**) to PKCδ Cys2 domain. The binding site of the receptor is shown using transparent surface area, and the H-bond is represented using dashed line; the blue spot is used to show the solvent exposure of the atoms for AAI (**B**) and ingenol mebutate (**D**), light green circles: hydrophobic amino acids; magenta circles: hydrophilic amino acids; blue circles: ligand exposure; and the darker color at AAI suggests that these atoms are more solvent exposed; and (**E**) Energetic contribution of residues at the binding site of PKCδ Cys2 domain to the enthalpy change of AAI (blue) and ingenol mebutate (red).

The binding modes of AAI and ingenol mebutate on PKCδ Cys2 domain (PDB code: 1PTR) [9] were very similar, with the cluster of hydroxyl groups oriented to the binding site and the cluster of methyl groups exposed to the solvent (Figure 6). Such orientation similarity between AAI and ingenol mebutate was not surprising in that the former only differentiates an acetyl group from the latter (Figure 6A,C). Despite such an orientation similarity, computational docking and molecular dynamics (MD) studies indicated that ingenol mebutate bound with the receptor more tightly and less solvent-exposed than AAI, and the acetyl group of AAI might blockade its further approaching to the binding pocket (Figure 6B,D). In addition, the hydroxyl group at ingenol mebutate formed two H-bonds with Leu23 and Thr12, whereas the acetyl group merely formed one H-bond with Thr12. The additional H-bond formed by ingenol mebutate might further strengthen its binding. To validate our observations from molecular docking, binding free energy calculation and decomposition were performed using the molecular mechanics poisson-boltzmann surface area (MM-PBSA) method [10]. As shown in Table 1, the G-free energy of ingenol mebutate was about 10 kcal/mol lower than that of AAI, supporting the above observations. However, our experimental studies demonstrated that the biological activity of AAI was comparable or even slightly better at low concentration than that of ingenol mebutate, which contradicted with the results from computational modeling. It was possible that the calculated free energy overestimated the experimental values, but it still could not explain the more favorable binding of ingenol mebutate than that of AAI observed in the modeling studies.

Thus, our theoretical calculations supported that the slightly improved biological activity of AAI might have originated from its favorable membrane penetration ability rather than its improved binding to PKCδ. Indeed, acetylation of the hydroxyl ingenol mebutate increases its LogP value from 2.33 to 2.9, making it more facile to penetrate cell membranes. It is likely that the acetyl group of AAI could be deprotected by the acetylase to form ingenol mebutate in the cell. Another possible reason for the enhanced bioactivity of AAI is the increased stability of AAI raised from the inhibition of the acyl migration from position 3 to position 20 due to the acetylation at position 20.

To further investigate the structure activity relationship between these two compounds, the PKCδ energy decomposition was performed using MM-PBSA. Energy decomposition indicated that the van der Waals interactions were the main driving force for the binding of AAI and ingenol mebutate, whereas the entropic component was not favorable for their binding (Table 1). In addition, the van der Waals contacts differentiated the binding affinity difference between AAI and ingenol mebutate, which suggested that improvement of the molecular surface fitting of ingenol mebutate to the binding pocket might strengthen its binding affinity to the receptor. Calculation of the energetic contribution for each of the residues of the binding site suggested that Met 9, Ser10, Pro11, Thr12, Leu20, Leu21, Trp22, Gly23, Leu24, and Qln27 were essential to the binding affinity of both AAI and ingenol mebutate (Figure 6E), and interactions with these residues should be considered for future compound modifications.

## 3. Discussion

A number of studies have shown that ingenol derivatives, such as ingenol mebutate, exhibited potent anti-HIV [11] and anti-cancer activities [12]. Here, we reported a synthetic ingenol derivative, AAI, whose only difference to ingenol mebutate was the acetylation in position 20. Due to the chemical structural similarity to ingenol mebutate, AAI bound to the same site in PKCδ in very similar binding modes. The biological activity of AAI was similar or even slightly better at low concentration than that of ingenol mebutate. Our results from computational modeling suggested that the better biological activity of AAI might have originated from its increased hydrophobicity rather than its improved binding affinity to the target protein PKCδ. Another possible reason for the better bioactivity of AAI was the increased stability of AAI raised from the inhibition of the acyl migration due to the acetylation at position 20. A previous report has shown the cytotoxicity of AAI against melanoma, breast cancer, and colon cancer [8]. In the expanded cytotoxicity evaluations, we found, in addition to the solid cancer cells, including MCF-7/ADR, HCT-116 and H1975, AAI was also sensitive to the leukemia cells, such as K562, HL-60, and KT-1 cells. Although AAI inhibited the cellular proliferation, however, unlike most of other chemical agents, the inhibition on the cell proliferation was not in a concentration-dependent manner. This concentration independence of AAI was similar to its parent compound ingenol mebutate [13,14], and the possible reason for this could be partly due to the toxic effect, as this kind of compounds could induce both necrosis and plasma membrane disruption, in addition to the apoptosis [13].

In the cellular model, we found that AAI arrested K562 cells at G2/M phase and induced mitochondrial apoptosis in K562 cell line, which is Bcr-Abl promoted chronic myeloid leukemia. Activation of AKT is one of the major signaling events in the Bcr-Abl leukemogenesis [15] and contributes to the development of drug resistance [16]. Moreover, other signaling molecules also play crucial roles in initiating and development of leukemogenesis. For example, activation of the JAK/STAT3 pathway also contributes to the initiating of the disease [17], and therefore JAK/STAT3 has been considered as an attractive therapeutic target for developing therapeutic agents. To further understand the mechanical basis of AAI-induced apoptosis, we investigated the signaling pathways known to regulate cell proliferation and survival. As Bcr-Abl activation is believed to be the initiating molecular event in chronic myeloid leukemia, we performed the kinase inhibition assay against Bcr-Abl. The results showed that AAI (0–20 μM) revealed no effect against the activity of Bcr-Abl, suggesting AAI inhibiting the growth of K562 cells was not via the inhibition of Bcr-Abl. Although AAI was not as effective as the clinical Bcr-Abl inhibitors, AAI was possibly effective against a novel critical target(s) other than Bcr-Abl, and provided an alternate selection of the treatment of chronic myeloid leukemia. In addition to Bcr-Abl, PKCs also regulate several signaling pathways that are crucial to leukemic cellular malignant transformation [18], and PKCδ has a close relationship with proliferation and apoptosis [14]. The parent compound ingenol mebutate has been shown to induce apoptosis in leukaemic cell lines, an effect that requires the expression of protein PKCδ. Chronic activation of PKCδ/ERK in leukemic cells delivers a pro-apoptotic, rather than a proliferative or survival, signal [14]. In the present study, we found that, as ingenol mebutate did, AAI also revealed excellent repeatability of its activation of PKCδ/ERK. Additionally, AKT activation is necessary and sufficient to inhibit apoptosis and induce transformation. In addition to the interaction with PKCδ [19,20], ingenol mebutate has been reported to regulate the AKT activity directly, which is considered as a non-PKC target for ingenol mebutate [14]. We also observed significant inhibitory effects of AAI against the activation of AKT. Therefore, like ingenol mebutate, AAI could also regulate these two potential targets and induce apoptosis in K562 cells. Furthermore, in addition to the reported effect on PKCδ and AKT, we found that AAI could inactivate the JAK/STAT3 pathway, which might be a new activity of ingenol derivatives. However, p-STAT2/5 remained unchanged under our present experimental conditions (Supplementary Materials, Figure S2). Bcr-Abl activates JAK/STAT [21,22], and AAI selectively inhibited the STAT3 pathway, but not STAT2/5, indicating that the inhibition of the STAT3 pathway was not due to the inhibition on Bcr-Abl, which further confirmed that AAI had no effect on Bcr-Abl. However, whether the effect on JAK/STAT3 pathways is related to the known targets, including PKCδ and AKT, needs further investigation. Since AAI could regulate multiple signaling molecules as presented in this work, it will be interesting to perform the global arrays to illustrate the changes in signaling pathways after AAI treatment in future studies

## 4. Materials and Methods

### 4.1. Drugs and Reagents

Ingenol mebutate was the product of Nanjing Spring and Autumn Biological Engineering Co., Ltd., Nanjing, China. Antibodies against survivin, AKT, p-AKT, ERK, p-ERK, p-PKCδ, PKCδ, p-JAK, JAK, p-STAT3, and STAT3, were purchased from Cell Signaling Technology, Beverly, MA, USA. Annexin V-FITC/PI apoptosis detection kit was provided by Nanjing KeyGEN BioTECH. Co., Ltd., Nanjing, China. Other reagents and kits were the products of Beyond, Nantong, China.

### 4.2. Gerneral Procedure for the Synthesis of 3-O-Angeloyl-20-O-acetyl ingenol

Compound **2**: Ingenol **1** (500 mg, 1.43 mmol) was dissolved in a solution of *p*-toluenesulfonic acid monohydrate (PTSA·H_2_O, 80 mg, 0.42 mmol) in acetone (1.5 mL). The solution was stirred at room temperature for 2 h (thin layer chromatography control). To this solution was added saturated aqueous solution of sodium hydrogen carbonate. Then, the obtained mixture was filtered, and the filtrate was concentrated in vacuo. The residue was taken up in ethyl acetate (10 mL). Finally, the mixture was concentrated and purified by chromatography (petroleum ether/ethyl acetate 5:1 to 3:1), giving 360 mg of the title compound **2**. The total yield was 64%.

Compound **3**: To a solution of compound **2** and angelic anhydride in THF was added a solution of LHMDS (lithium hexamethyldisilazide) in THF (0.1 mol/L) at 15 °C. The solution was stirred at room temperature for 40 min. The reaction was quenched by saturated aqueous solution of NH_4_Cl and the mixture was extracted by ethyl acetate, and then the organic layer was washed with brine and dried by Na_2_SO_4_. The organic layer was finally concentrated and purified by chromatography (petroleum ether/ethyl acetate 10:1 to 5:1) to give product **3**, as a white solid (115 mg, 95%).

Compound **4**: A solution of compound **3** (50 mg, 0.11 mmol) in 1% HCl (MeOH) was stirred at 25 °C for 1 h. The mixture was concentrated in vacuo at room temperature. The residue was suspended in water and extracted with ethyl acetate. The organic layer was washed with brine, dried by Na_2_SO_4_, and concentrated to give the crude product as a colorless solid (45 mg, 98%).

Compound **5**: To a solution of compound **4** (100 mg, 0.23 mmol) in pyridine was added Ac_2_O (25 mg, 0.24 mmol) at 10 °C and the reaction mixture was stirred at room temperature overnight. The mixture was extracted with ethyl acetate and the organic layer was washed by HCl (1 M), water and saturated aqueous solution of NaHCO_3_ and brine, dried by Na_2_SO_4_. The mixture was concentrated and purified by chromatography (petroleum ether/ethyl acetate 10:1 to 5:1) to give the product **5** as a white solid (90 mg, 82%).

### 4.3. Cell Lines and Cell Culture

A549, HCT-116, HeLa, and MCF-7/ADR cell lines were obtained from the American Type Culture Collection (Manassas, VA, USA). K562, HL-60, KT-1, HIN-3T3, L-02, and H1975 cell lines were provided by the Cell Bank of the Chinese Academy of Sciences (Shanghai, China). A549 cells were cultured in F-12K medium with 10% fetal bovine serum (FBS). HeLa, HIN-3T3, and HCT-116 cells were cultured in Dulbecco’s Modified Eagle’s Medium with 10% FBS. The others were cultured in Roswell Park Memorial Institute (RPMI) 1640 medium with 10% FBS.

### 4.4. Cell Proliferation Inhibition Assay

The effect on cell proliferation was evaluated by the MTT method. In general, cells were incubated with different concentrations of AAI or ingenol mebutate at indicated time. MTT solution (20 μL, 0.5 mg/mL) was added in the cell culture medium and incubated for another 4 h. Finally, the dye crystals were dissolved with dimethyl sulfoxide, and the absorbance was measured at 570 nm. The effect on the cell proliferation was expressed as inhibition rate.

### 4.5. Cell Cycle Distribution Assay

The K562 cells were treated with certain concentration of AAI for indicated time. After AAI-treatment, the cells were collected, washed, and fixed with ice-cold 70% (*v*/*v*) ethanol at −20 °C for 24 h. The samples were resuspended and then stained with PI (50 μg/mL) containing RNase (10 μg/mL) for 20 min. Finally, the cell cycle distribution was analyzed with flow cytometry (Bection Dickinson, Waltham, MA, USA).

### 4.6. Apoptosis and Necrosis Assay by Annexin V-FITC/PI Double Staining

The stimulation of apoptosis and necrosis by AAI was assayed by Annexin V-FITC/PI double staining kit. K562 ells (5 × 10^5^) were treated with AAI (0–500 nM) for 18 h, and then harvested, washed, and resuspended in binding buffer. Double staining was started by adding Annexin V-FITC and PI, 5 μL, respectively, and incubating in the dark for 10 min. After staining, the samples were analyzed with flow cytometry (Bection Dickinson, Waltham, MA, USA).

### 4.7. MMP Assay

MMP of K562 cells was detected according to the manufacture’s instructions in the kit (Beyond, Nantong, China). Briefly, K562 cells were incubated with different concentrations of AAI (0–500 nM) or different time periods. After the treatment, cells were stained by JC-1 for 20 min at 37 °C. MMP were analyzed by flow cytometry. JC-1 was excited at 488 nm. Emissions at 535 nm and 595 nm were used to quantify the mitochondria with JC-1 green monomers and JC-1 red aggregate fluorescence, respectively. Frequency plots were prepared for FL1 and FL2 to determine the percentage of mitochondria stained green and red.

### 4.8. Western Blotting Assay

K562 cells were treated with AAI for the indicated time and concentrations, and then cells were lysed with RIPA buffer and total proteins were obtained. Protein samples were resolved on sodium dodecyl sulfate polyacrylamide gel electropheresis (SDS-PAGE, 10%–15%), and transferred to nitrocellulose membranes. The proteins were probed with primary antibodies, and followed by incubation with secondary antibodies. Finally, the bands were detected by enhanced chemiluminescence.

### 4.9. Computational Docking

Molecular docking was performed using molecular operating environment software (MOE, chemical computing group, Quebec City, QC, Canada) with AMBER12:EHT force field [23]. AAI and ingenol mebutate were drawn using the molecular scratch module in MOE, and then they were minimized using 10,000 steps of steepest minimization. For the docking studies, the crystal structure of the cys2 activator-binding domain of PKCδ in complex with phorbol ester (PDB code: 1PTR) was obtained from the protein databank (http://www.rcsb.org). The induced fit docking approach was applied for consideration of the flexibility of the side chains of the residues at the binding site. The produced conformation with the best score was selected for the analysis.

### 4.10. MD Simulations

MD simulations were carried out on PKCδ in complex with AAI and ingenol mebutate, respectively, using the Amber 12 package (University of California, Oakland, SF, USA). General AMBER force field and FF03 force field were employed for the ligand and the receptor, respectively. Prior to the MD simulations, the complex was solvated into an octagon box of TIP3P water molecules and neutralized using Na^+^. Then, it was minimized to remove unfavourable van der Waals interactions. The minimization consisted of two steps. First, only the water molecules and ions were minimized with 1000 steps of steepest descent minimization and 1000 steps of conjugate gradient minimization. Second, the restraint on the solute was removed and the whole system was relaxed with 3000 steps of steepest descent minimization and 3000 steps of conjugate gradient minimization. The cutoff of the non-bonded interactions was set to 12 Å for the energy minimization process. After minimization, MD was performed. First, the solute was restrained and the whole system was gradually heated from 10 to 300 K in 100 ps in the NVT (keep the number of molecules, volume and temperature constant) ensemble. Then the system was equilibrated in the NPT ensemble where the temperature and pressure were kept at 300 K and 1 atm, respectively. Finally, in the production process, the whole system was relaxed except for two cluster of residues bound with Zn^+2^ and a 30-ns molecular dynamics process was carried out. The two Zn^2+^ were far from the binding site and covalently bound with two clusters of residues. To simulate the covalent binding effect, we applied for a soft restraint on the two clusters of residues. For all MD steps, we set the time step to 0.002 ps, and the particle mesh Ewald (PME) method [24] was applied to deal with long-range electrostatic interactions; the lengths of the bonds involving hydrogen atoms were fixed with the SHAKE algorithm, as described previously [25].

### 4.11. Binding Energy Calculations

Binding free energy calculation and decomposition were performed using the MM-PBSA script in AMBER 12, as described previously [26]. Parameter setup was the same as our previous studies [26].

### 4.12. Data Analysis

One-way ANOVA with Tukey’s post hoc test was used for statistical analysis of the data, and values were expressed as mean ± SD. Differences of *p* < 0.05 were considered statistically significant.

## 5. Conclusions

In summary, the present study reported that ingenol mebutate acetylated derivative AAI displayed more potent in vitro cytotoxicity than ingenol mebutate at very low concentrations. AAI induced G2/M phase arrest, exhibiting potent apoptotic and necrotic activity in K562 cells. The results of molecular mechanism studies showed that AAI stimulated the activation of PKCδ and ERK, inhibited the activation of AKT, and inhibited JAK/STAT3 signaling pathways. These studies revealed that AAI had cytotoxic effects in different cell lines, and could be developed as an alternate therapeutic agent for solid cancer and leukemia.

## Figures and Tables

**Table 1:** Decomposition of free energy of AAI and ingenol mebutate to PKCδ Cys2 domain.

Ligand	Free Energy (kcal/mol)					
	VDW	EEL	EGB	ESURF	TΔS	ΔG
AAI	−30.69 (0.39)	−15.80 (0.68)	25.14 (0.41)	−3.95 (0.04)	−20.39 (2.86)	−4.91 (2.54)
ingenol mebutate	−40.51 (0.34)	−19.20 (0.48)	27.91 (0.27)	−4.46 (0.02)	−20.31 (2.71)	−15.95 (2.31)

VDW, van der Waals contribution from MM; EEL, electrostatic energy as calculated by the MM force field; EGB, the electrostatic contribution to the solvation free energy calculated by GB; ESURF, nonpolar contribution to the solvation free energy calculated by an empirical model.

## Supplementary Materials

Supplementary materials can be found at www.mdpi.com/1422-0067/17/8/1348/s1.
