# Supplementary material for: Synthesis and Cytotoxicity against K562 Cells of 3-O-Angeloyl-20-O-acetyl Ingenol, a Derivative of Ingenol Mebutate
Source: Int J Mol Sci. 2016 Aug 19;17(8):1348. doi: 10.3390/ijms17081348 (PMC5000744; doi:10.3390/ijms17081348)
Supplement: Supplementary file 1 [file ijms-17-01348-s001.pdf]

Supplementary Materials: Synthesis and Cytotoxicity against K562 Cells of 3-*O*-Angeloyl-20-*O*-acetyl Ingenol, a Derivative of Ingenol Mebutate

Ming Liu, Fangling Chen, Rilei Yu, Weiyi Zhang, Mei Han, Fei Liu, Jing Wu, Xingzeng Zhao and Jinlai Miao

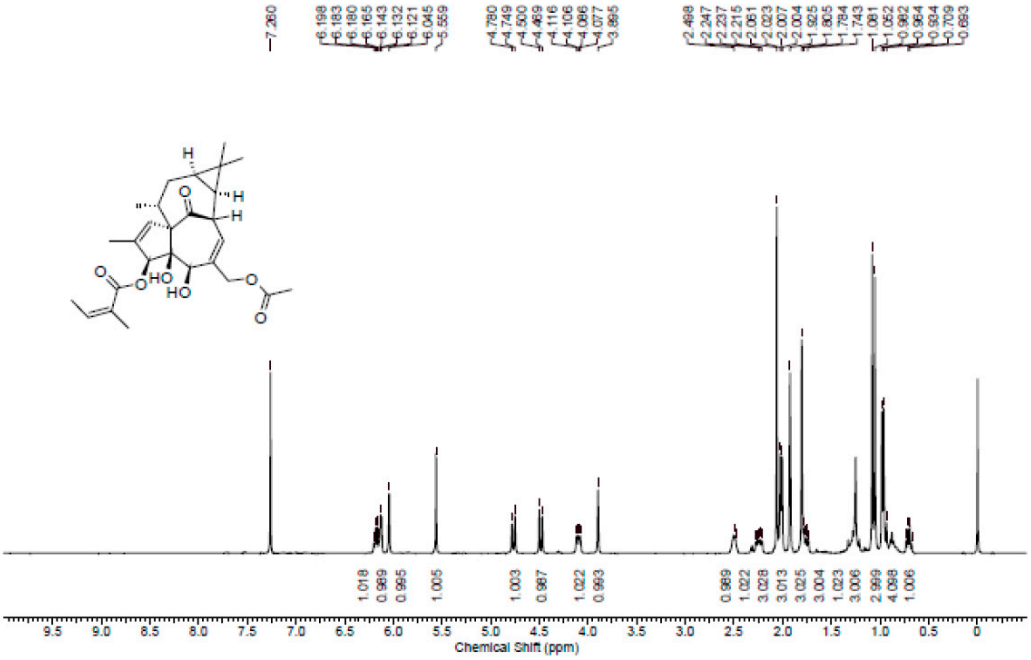

Figure S1. ^{1}H-NMR spectra for 3-*O*-angeloyl-2-*O*-acetyl ingenol (AAI).

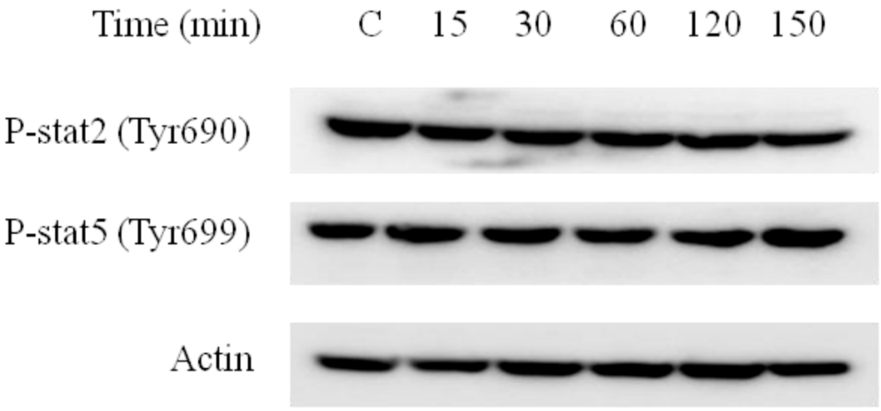

Figure S2. AAI does not affect the activation of stat2 and stat5.
